# Effect of Dispersion Method on Stability and Dielectric Strength of Transformer Oil-Based TiO_2_ Nanofluids

**DOI:** 10.1186/s11671-016-1738-5

**Published:** 2016-11-24

**Authors:** Yu-zhen Lv, Chao Li, Qian Sun, Meng Huang, Cheng-rong Li, Bo Qi

**Affiliations:** 1 School of Energy, Power and Mechanical Engineering, North China Electric Power University, Beijing, 102206 China; 2 State Key Laboratory of Alternate Electrical Power System with Renewable Energy Sources, North China Electric Power University, Beijing, 102206 China; 3 Beijing Key Laboratory of High Voltage and EMC, North China Electric Power University, Beijing, 102206 China

**Keywords:** Dispersion method, Functionalized TiO_2_ nanoparticle, Nanofluid, Stability, Breakdown strength

## Abstract

Dispersion stability of nanoparticles in the liquid media is of great importance to the utilization in practice. This study aims to investigate the effects of mechanical dispersion method on the dispersibility of functionalized TiO_2_ nanoparticles in the transformer oil. Dispersion methods, including stirring, ultrasonic bath, and probe processes, were systematically tested to verify their versatility for preparing stable nanofluid. The test results reveal that the combination of ultrasonic bath process and stirring method has the best dispersion efficiency and the obtained nanofluid possesses the highest AC breakdown strength. Specifically, after aging for 168 h, the size of nanoparticles in the nanofluid prepared by the combination method has no obvious change, while those obtained by the other three paths are increased obviously.

## Background

Nanofluids are a new type of engineering materials by dispersing nanoparticles into the base fluid, which have received considerable attention for many years due to their superior thermal and dielectric properties [1–4]. Transformer oil, as a cooling and insulating medium, is a major part of the electrical insulation system in many types of electrical equipment, such as transformers, cables, and bushings. The dielectric strength and thermal conductivity of transformer oil are of great importance to keep power transformers operating safely and optimize their structure design. Recently, it has been found that the presence of nanoparticles can greatly improve thermal conductivity and breakdown strength of transformer oil [5–12]. However, the nanoparticles tend to aggregate into bigger particles mainly by the attractive forces and external stresses [11–13], leading to the performance degradation of nanofluids. So, the long-term stability of nanoparticles dispersion in the host oil is still a key challenge in this field.

Much work has been done to improve the dispersion stability of the nanoparticles in the base fluid [14–16]. In comparison with mechanical method, surface functionalization has been proved to be a more useful approach to control the balance of Van der Waals attraction and electrostatic repulsion between nanoparticles through surface modification of nanoparticles [17, 18]. Dispersion stability of iron oxide nanoparticles in mineral oil was greatly improved by optimizing their surface functionalization state and the nanofluid had no visible sedimentation after aging for 24 months at room temperature [17]. The dispersion of TiO_2_ nanoparticles in the mineral oil can also be improved by adjusting the usage of modifying agents to functionalize the nanoparticles [18]. Meanwhile, the adsorption of functional groups on the surface of nanoparticles could be influenced by many factors, including temperature, the type of base liquid, and the interaction between functional group and nanoparticle. Although the mechanical dispersion process can provide energy to overcome the adhesion force between nanoparticles, it may affect the interaction between functional group and nanoparticle at the same time. However, no other studies have been found directly point out the effect of the mechanical dispersion method on the stability and breakdown strength of transformer oil-based nanofluids modified by functionalized nanoparticles.

In this paper, TiO_2_ nanoparticles functionalized by oleic acid were synthesized by a solvothermal method. Three kinds of mechanical dispersion methods were employed to prepare transformer oil-based TiO_2_ nanofluids. The dispersion stability, AC breakdown strength, and thermos-physical property of obtained nanofluids were measured and compared.

## Methods

### Nanoparticle Synthesis and Functionalization

TiO_2_ nanoparticles were prepared and functionalized by using titanium n-butoxide and DI water as reactants by a solvothermal method. In a typical procedure, reactants were first introduced into a mixed solution of cyclohexane and triethylamine under stirring. After stirring for 5 min, oleic acid was added into the above solution at room temperature with vigorous agitation. The resulting mixture was subsequently heated to the temperature of 150 °C. After heating for 24 h, the resulting product was cooled down naturally and washed with distilled water and absolute ethanol for several times to remove the ions possibly remaining in the product and finally dried in the vacuum at 70 °C.

### Nanofluid Preparation

**Fig. 1:**
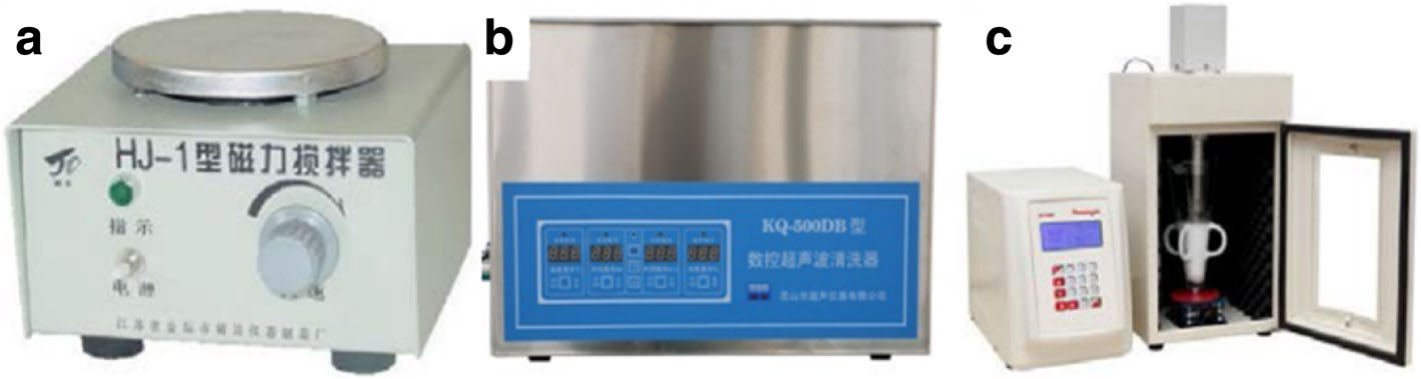
Images of dispersion instruments. **a** Magnetic stirrer. **b** Ultrasonic bath. **c** Probe

In order to study the effect of dispersion method on the stability of nanofluid, functionalized TiO_2_ nanoparticles with the same volume fraction of 0.075% were added into the mineral transformer oil (No. 25 Karamay). After treating for 3 min in ultrasonic bath, the obtained mixture was then divided into 16 parts. The three kinds of dispersion instruments are shown in Fig. 1. The six parts were stirred using a magnetic stirrer for a time range from 10 to 180 min at a rotate speed of 1800 r/m. The other six parts were placed in an ultrasonic bath and treated for the same times as that of stirring method at 20 kHz. Four parts were sonicated by a probe for 10, 20, 30, and 40 min at 20 kHz, respectively. To avoid overheating, the mixtures were ultra-sonicated for every 5 min by a break duration about 1 min.

### Characterization Method

The morphology of the as-prepared nanoparticles was characterized by high-resolution transmission electron microscopy (HRTEM: JEM-2100F). Fourier transform infrared spectra (FT-IR) was used to analyze the surface functionalization state of TiO_2_ nanoparticles scanned from 400 to 4000 cm^-1^ with a resolution of 4 cm^-1^. A dynamic light scattering device (Malvern Nano ZS90) was used to determine the average size of nanoparticles in the fresh and aged nanofluids. The polydispersity index (PdI) describes the width of the particle size distribution. The viscosity of pure oil and nanofluid was measured at the temperature of 29 °C with the rotational viscometer Brookfield DVII, and their thermal conductivity was characterized by a Netzsch LFA447 tester. A portable Jian-tong Oil Tester 6801 was used to measure AC breakdown voltages of pure oil and nanofluids according to IEC standard 60156 using brass spherically capped electrodes set at 2 mm gap.

## Results and Discussion

### Morphology and Surface Functionalization of Nanoparticles

**Fig. 2:**
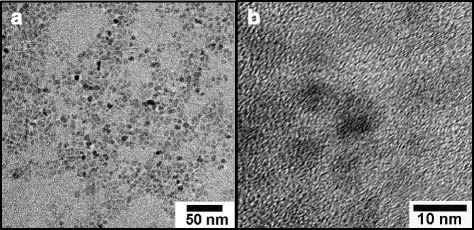
Morphology of as-prepared TiO_2_ nanoparticles (**a**) TEM image of many nanoparticles (**b**) HRTEM image of monodisperse nanoparticles

The morphology of the as-prepared TiO_2_ nanoparticles is shown in Fig. 2. It can be clearly seen that the as-prepared nanoparticles have a small average diameter of 6 nm and exhibit a uniform particle size distribution. No obvious aggregation was observed among the nanoparticles. Moreover, the clear lattice fringes of single nanoparticles in Fig. 2b demonstrate the single-crystalline nature of the nanoparticles.

**Fig. 3:**
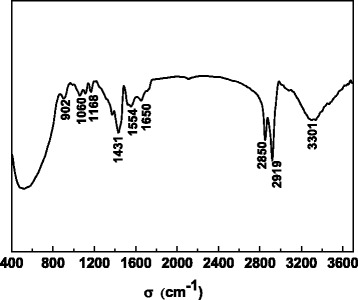
FT-IR spectrum of as-prepared TiO_2_ nanoparticles

The FT-IR spectrum of as-prepared TiO_2_ nanoparticles is depicted in Fig. 3. The IR peak around 500 cm^-1^ is attributed to the TiO_2_, whereas the absorption peaks related with the functional group of oleic acid are at higher bands [19, 20]. The transmission bands at 3301 and 1060 cm^-1^ are due to the presence of hydroxyl group (–OH). The bands in the 2919 and 2850 cm^-1^ region are associated with the asymmetric and symmetric –CH_2_– and –CH_3_ modes of the oleic acid-saturated chain fragments. The extra peaks around 902 and 1168 cm^-1^ can be assigned to stretching vibration of –C–O– groups [21]. It should be note that the peak at 1717 cm^-1^ associated with the C=O stretching mode is not observed in the spectrum [22]. This means that no free physically absorbed oleic acid exists in the nanoparticles.

The C=O stretch from oleic acid is replaced by the appearance of two new peaks at 1554 and 1431 cm^-1^, which correspond to the asymmetric and symmetric carboxylate (–COO–) stretching modes [23, 24]. Studies have shown that these two peaks could be utilized to predict the types of binding interaction between the carboxylate head and metal oxide surface [25]. Depending on the wave number separation between asymmetric and symmetric peaks, it is indicated that the functional group of oleic acid is covalently bonded with the titanium sites at the nanoparticles surface mainly by bidentate linkages [17, 26]. These results confirm that the carboxylate group is chemically bonded with the surface titanium ion, and the as-prepared TiO_2_ nanoparticles are well functionalized by oleic acid.

### Dispersion Stability of Nanofluids

**Fig. 4:**
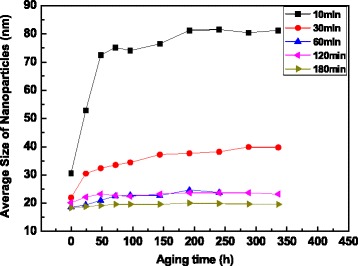
Nanoparticle size vs. aging time with a variety of stirring times

The effect of the stirring time on the average size of TiO_2_ nanoparticles in the nanofluids are shown in Fig. 4. It can be seen that the nanoparticle size in the fresh nanofluid is dropped down with the increasing of stirring time.

For the nanofluid prepared by stirring for 10 min, the nanoparticle size is abruptly enlarged from 30.63 to 72.52 nm with the prolonging of aging time and then achieves a constant value of 81.24 nm after aging for 192 h. With the increasing of stirring time from 30 to 180 min, the stability of as-prepared nanofluids is greatly improved. The size of nanoparticles in nanofluid stirring for 180 min is smaller than those in other nanofluids, which is in the range from 18.3 to 20.0 nm during the aging time of 336 h. In addition, the PdI of particles in this nanofluid is 0.16, indicating the uniform size distribution of nanoparticles. These test results demonstrate that for the functionalized TiO_2_ nanoparticles, the shear force at a high agitation speed can decrease the tendency of particle agglomeration and the nanofluid with a good dispersion stability can be observed by stirring for 180 min.

**Fig. 5:**
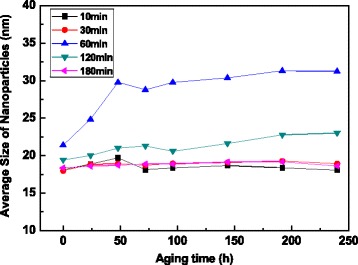
Nanoparticle size vs. aging time with a variety of dispersion times of ultrasonic bath

The variation of nanoparticle size in the nanofluids with the aging time by the ultrasonic bath processing is studied and shown in Fig. 5. The nanoparticle size in the fresh nanofluids keeps in the range of 18.1 to 21.4 nm with the increasing of ultrasonic treating time, which are much smaller than those obtained by the stirring method. This indicates that the ultrasonic bath dispersion is more efficient to prepare well-dispersed nanofluid due to its uniform higher intensity energy input. With the prolonging of treatment time from 30 to 60 min, the nanoparticle size is obviously increased and then decreased when the treatment time is prolonged from 120 to 180 min. This changing tendency is totally different with that observed in stirring dispersion process. It is well-known that the collapse of cavitation bubbles under ultrasonic treatment can release and transfer a good deal of energy into the nanofluid, which greatly decrease the agglomeration of nanoparticles. Meanwhile, the temperature of the nanofluid can be remarkably increased with a long-time treatment. This rise of the temperature in nanofluid will influence the adsorption equilibrium of functional groups on the surface of nanoparticles. By treating for 60 min, the rise of temperature probably makes the functional group begin to detach from the nanoparticles and has no enough time to re-attach, finally leading to the agglomeration of nanoparticles. This desorption tendency of functional group is inhibited due to the functional group reacts with the surface of nanoparticles when the treating time is prolonged to 120 and 180 min. In all, the nanofluid obtained by ultrasonic bath treatment for 10 min has the best dispersion stability and its nanoparticle size keeps in the range of about 18.1 nm with a PdI value of 0.26 even after aging for 240 h.

**Fig. 6:**
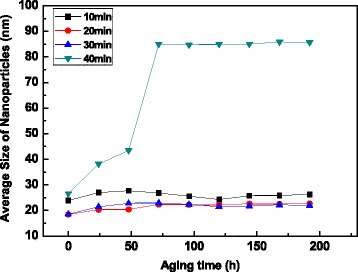
Nanoparticle size vs. aging time with a variety of dispersion times of ultrasonic probe

The average size of nanoparticles in the nanofluids ultra-sonicated with the probe is shown in Fig. 6. It can be clearly seen that the sizes of nanoparticles in fresh nanofluids are decreased from 23.8 (10 min) to 18.4 nm (20 and 30 min) first and then increased to 26.5 nm after treated for 40 min, which is 5.7 nm larger than that obtained by the ultrasonic bath process after treating the same time of 10 min. The nanoparticle size in nanofluid treated for 40 min is abruptly increased to 85 nm and maintained this value after aging for 192 h, while those treated for 20 and 30 min maintain below 23 nm and exhibit good stability with a PdI value of 0.31. This value is still 4.9 nm higher than that obtained by the ultrasonic bath method, and the uniformity of size distribution is also lowered. Although the ultrasonic probe process provides the higher energy to the suspension, this high-intensity energy is limited around the tip due to its small diameter. With the prolonging of the treatment time, the temperature of the nanofluid tends to greatly increase and the molecules of functional group on the surface of nanoparticles have a tendency to decompose into the oil. Then nanoparticles are much easier to agglomerate with each other, and the stability of nanofluid is getting worse. During the ultrasonic bath process, a more uniform high-intensity sonication energy is provided and its heating rate is much slower than that in the ultrasonic probe process. Therefore, the average sizes of nanoparticles treated with the bath are significantly smaller than those of the probe treated at the same treating time.

**Fig. 7:**
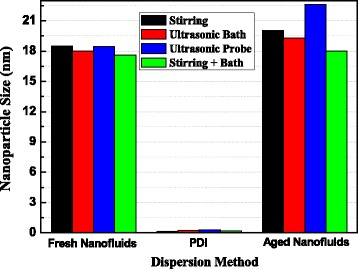
Nanoparticle size vs. dispersion method in the fresh and aged nanofluids for 168 h

Based on the obtained results, we can see that the stirring and ultrasonic bath processes show better dispersion efficiency than ultrasonic probe process. So, the combination of these two methods under their optimum condition was used to prepare nanofluid with the same loading of functionalized nanoparticles. The average size of nanoparticle in the obtained fresh nanofluid and aged nanofluid for 168 h is shown in Fig. 7 and compared with those obtained by other three methods.

**Fig. 8:**
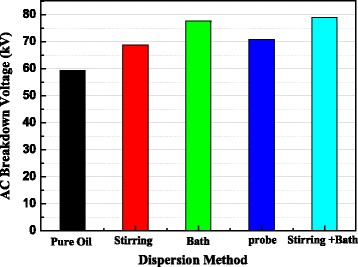
AC breakdown voltage for pure oil and nanofluids vs. dispersion method

After stirring for 180 min and ultra-sonicated for 10 min in bath, the size of nanoparticles in the nanofluid is 17.6 nm, smaller than those obtained by each method individually. Specifically, after aging for 168 h, the size of nanoparticles in the nanofluid prepared by the combination method has no obvious change, while those obtained by the other three paths are increased obviously. The AC breakdown voltages of four kinds of fresh nanofluids were tested and compared with that of pure oil. As shown in Fig. 8, all the AC breakdown strength of nanofluids are higher than that of the pure oil and the nanofluid obtained by the combination dispersion method is improved by 32.8%, possessing the highest breakdown performance.

The thermal conductivity and viscosity of pure oil and nanofluid prepared by the combination method is measured and shown in Table 1. As can be seen, the TiO_2_ nanofluid shows no obvious improvement compared to the pure oil. The thermal conductivity of nanofluid is only increased by 1.2%. It is considered that thermal conductivity of nanofluids is mainly related with the physical nature of base liquid and nanoparticles, the diameter and concentration of nanoparticles. About 10% enhancement in thermal conductivity of lubricant-based nanofluids has been reported by the presence of 0.25 wt% TiO_2_ nanoparticles [6]. It is considered that the enhancement on the thermal conductivity is due to the formation of clusters by agglomerated nanoparticles, which provide channels for thermal waves and transport of heat [6, 7]. However, the volume fraction of TiO_2_ nanoparticles in our nanofluid is only 0.075% and the size of nanoparticles keeps uniform around 18 nm. The effects of concentration of TiO_2_ nanoparticles and their agglomeration state on the thermos-physical property of transformer oil-based nanofluids are under study.

**Table 1:** Thermal conductivity and viscosity for the transformer oil and nanofluid

Oil sample	Thermal conductivity W/(m K)	Viscosity (29 °C) mPa s
Transformer oil	0.3714	12.4
Nanofluid	0.3758	12.5
Enhancement ratio (%)	1.2	0.8

## Conclusions

This study investigated the dispersion stability of functionalized TiO_2_ nanoparticles in the transformer oil-based nanofluids, their AC breakdown strength and thermos-physical property, which were prepared through stirring, ultrasonic bath, and probe processes. The test results show that the dispersion stability of functionalized nanoparticles is clearly dependent on the dispersion method. The stirring and ultrasonic bath processes exhibit better dispersion efficiency than the ultrasonic probe process, which may disrupt the adsorption balance of functional group on the surface of nanoparticles due to the limitation of the high-intensity sonication energy around the probe tip. The combination method of stirring and ultrasonic bath can effectively reduce the tendency for nanoparticles to agglomerate and prepare the nanofluid with the best dispersion stability and breakdown performance.
